# Delirium and Iatrogenic Withdrawal Profiles in the Pediatric Intensive Care Unit (PICU): A Latent Profile Analysis of Nurse-Administered Bedside Scales

**DOI:** 10.3390/jcm15166134

**Published:** 2026-08-07

**Authors:** Yujiro Matsuishi, Haruhiko Hoshino, Yuki Enomoto, Takahiro Kido, Nobutake Shimojo, Mitsuki Ikeda, Misaki Kotani, Bryan J. Mathis, Yoshiaki Inoue

**Affiliations:** 1 Women’s Health Nursing and Midwifery, Nursing Science and Engineering, Institute of Medicine, University of Tsukuba, Tsukuba 305-8577, Japan; 2 Department of Nursing, Faculty of Medical Technology, Teikyo University, Tokyo 192-0395, Japan; 3 Department of Emergency and Critical Care Medicine, Institute of Medicine, University of Tsukuba, Tsukuba 305-8577, Japan; 4 Department of Pediatrics, University of Tsukuba Hospital, Tsukuba 305-8577, Japan; 5 International Medical Center, University of Tsukuba Hospital, Tsukuba 305-8577, Japan

**Keywords:** intensive care units, pediatric, delirium, substance withdrawal syndrome, nursing assessment, sedation, analgesia, latent profile analysis, SOS-PD

## Abstract

**Background/Objectives:** Delirium and iatrogenic withdrawal syndrome (IWS) overlap clinically in critically ill children. We used latent profile analysis (LPA) of nurse-administered assessments to identify score patterns, not diagnoses; no independent reference standard was available. **Methods:** This single-center retrospective cohort included 203 pediatric intensive care unit (PICU) records from 84 patients. Raw scores from the IWS and delirium subscores of the Japanese Sophia Observation withdrawal Symptoms–Paediatric Delirium (SOS-PD) scale, the Richmond Agitation–Sedation Scale (RASS), and the Face, Legs, Activity, Cry, Consolability (FLACC) scale were modeled using tied-diagonal Gaussian mixtures for K = 1–5. Selection used the Bayesian information criterion (BIC), entropy, and posterior probabilities. **Results:** The selected four-profile solution had BIC = 3245.4, entropy = 0.887, and 91.6% of records had a maximum posterior probability ≥ 0.70. Patient-level analyses provided partial support (adjusted Rand index = 0.833); standardization yielded an adjusted Rand index of 0.977. The profiles were Profile 1 (Delirium-predominant), Profile 2 (High-symptom mixed), Profile 3 (Withdrawal-predominant), and Profile 4 (Low-symptom at-target). Separation was strongest and inverse on the SOS-PD subscores (within-input |d| = 3.05 withdrawal; 2.26 delirium), describing input-scale divergence rather than diagnostic discrimination. Untied, categorical, and count models favored two profiles. **Conclusions:** Joint bedside scores contained model-specific candidate patterns, and profile number was model-dependent. Before prospective external validation, profiles should be treated only as descriptive score summaries and should not be used in clinical care.

## 1. Introduction

Pediatric intensive care unit (PICU) nurses routinely assess sedation, pain, delirium, and iatrogenic withdrawal syndrome (IWS). These interrelated problems are associated with prolonged ventilation, longer intensive care stay, and adverse neurodevelopmental outcomes; pediatric delirium is also associated with brain-injury biomarkers after cardiac surgery [1]. The European Society of Paediatric and Neonatal Intensive Care (ESPNIC) and 2022 pediatric guidelines therefore recommend nurse-led multidomain monitoring [2,3]. However, separate interpretation of each scale can obscure overlapping signals.

Delirium and sedative- or hypnotic-related IWS share irritability, agitation, sleep–wake disturbance, motor restlessness, and autonomic activation. Pediatric tools therefore lack specificity at this boundary [4]. Previous studies predicted IWS [5] or derived delirium subtypes [6,7], but did not jointly differentiate pediatric delirium and IWS. Existing delirium tools—the Cornell Assessment of Pediatric Delirium (CAPD) [8], Pediatric Confusion Assessment Method for the Intensive Care Unit (pCAM-ICU) [9], Preschool Confusion Assessment Method for the Intensive Care Unit (psCAM-ICU) [10,11], and Sophia Observation withdrawal Symptoms–Paediatric Delirium (SOS-PD) scale [12]— screen for delirium but do not resolve coexisting withdrawal.

Latent profile analysis (LPA) uses Gaussian mixture modeling to derive profiles from multiple scales. Related analyses identified outcome-associated acute respiratory distress syndrome (ARDS) [13] and sepsis [14] subphenotypes, supporting multidimensional profiling [15]. Unlike those externally validated prognostic phenotypes, our profiles are described using the same nurse-rated scales from which they were derived and have no established prognostic or treatment-response validity.

We used LPA to identify multidimensional bedside profiles of sedation, analgesia, delirium, and IWS. We hypothesized distinct withdrawal- and delirium-predominant patterns. Because the same subscores define and describe the profiles, this analysis characterizes indicator co-variation rather than diagnostic differentiation. It generates model-specific candidate profiles for future validation of the gap identified by Madden et al. [4].

## 2. Materials and Methods

### 2.1. Study Design and Setting

This was a single-center retrospective observational cohort study conducted in the eight-bed mixed-use post-surgical and internal-medicine tertiary PICU at the University of Tsukuba. The unit of analysis was the individual nurse-completed bedside assessment record obtained during a PICU admission. Reporting follows the Strengthening the Reporting of Observational Studies in Epidemiology (STROBE) recommendations [16]; a completed Guidelines for Reporting on Latent Trajectory Studies (GRoLTS) checklist with longitudinal-trajectory items marked not applicable to this cross-sectional design is provided in Supplementary Note S3.

### 2.2. Participants and Selection

All consecutive PICU patients assessed during routine multidisciplinary team rounds with the Japanese SOS-PD were screened for eligibility. Records were collected between October 2018 and December 2020. Following the protocol of the linked validation study [17,18], assessments were administered during scheduled weekly team rounds, with minor adjustments only for urgent clinical events. A patient could contribute more than one record if their stay spanned multiple rounds; an assessment record denotes one weekly team-round assessment of one patient.

Records were excluded for unarousable status throughout observation (Richmond Agitation–Sedation Scale [RASS] ≤ −4), because arousal-dependent items could not be scored, or when structural neurological disorder or developmental encephalopathy precluded behavioral assessment. The linked validation-study age limits were gestational age ≥ 40 weeks and chronological age ≤ 20 years [17]. Hierarchical application of criteria assigned each exclusion one reason (Section 3). Patient identifiers obtained after the primary analysis enabled adjusted and robustness analyses (Section 3; Supplementary Note S1). Assessment records were the analysis unit because they represented bedside states rather than fixed patient attributes.

### 2.3. Bedside Assessment Variables

Three nurse-administered instruments provided four inputs. The 22-item Japanese SOS-PD [12], derived from the Sophia Observation withdrawal Symptoms (SOS) scale [19], provided separate IWS and delirium subscores. RASS assessed sedation–agitation [20] and was retained to characterize profiles. The Face, Legs, Activity, Cry, Consolability (FLACC) scale assessed pain [21]; its Japanese version is validated [22]. SOS-PD-IWS, SOS-PD delirium, RASS, and FLACC entered the model on their raw scales. All 203 records were 100% complete for these inputs; no imputation was required.

### 2.4. Latent Profile Model

LPA, or latent class analysis with continuous indicators, grouped records by four bedside scores using a Gaussian mixture model. Each record was assigned to its highest probability profile. A prespecified equal-volume, equal-shape diagonal covariance structure (mclust EEI) allowed profile means to differ while sharing parsimonious within-profile variances. Raw scales preserved interpretation in the instruments’ original units.

### 2.5. Class Enumeration and Selection

We estimated K = 1 through K = 5 and selected the class number using the Bayesian information criterion (BIC; lower is better), entropy (≥0.80 adequate), and the proportion with maximum posterior probability ≥ 0.70 [15,23,24,25]. The bootstrap likelihood ratio test (BLRT; 999 replicates) was supplementary because it may over-extract with finite samples [24,26] (Supplementary Note S2). Modal posterior probability was used to assign classes; if criteria converged, the smaller model was retained [24,25]. Post hoc labels describe dominant score patterns: Profile 1 (Delirium-predominant), Profile 2 (High-symptom mixed), Profile 3 (Withdrawal-predominant), and Profile 4 (Low-symptom at-target). Numbers remain primary because profiles are not validated diagnoses.

### 2.6. Class Descriptions and Group Comparisons

Class profiles were summarized as mean ± standard deviation (SD) for each assessment scale, and PICU length of stay as median [interquartile range (IQR)]. Between-class separation was quantified with η^2^ and pairwise Cohen’s d, reported as absolute values |d|. Continuous variables were compared across classes with the Kruskal–Wallis test and pairwise Mann–Whitney U tests; binary medication and respiratory-support variables with omnibus 4 × 2 χ^2^ tests followed by pairwise Fisher exact tests, Bonferroni-corrected across the pairwise binary contrasts, with both uncorrected and corrected *p* values reported. Test statistics, effect sizes, and the per-comparison threshold are in Supplementary Note S2 and Table S3.

### 2.7. Adjusted Mixed-Effects Models

Two prespecified mixed-effects model families adjusted medication associations for age, post-operative status, developmental delay, trisomy, respiratory support, and within-patient clustering via random intercepts. One-vs-rest logistic generalized linear mixed models (GLMMs) modeled profile membership, while cumulative link mixed models (CLMMs) modeled bedside-score categories; as such, their odds ratios indicate higher-category proportional odds (Supplementary Note S2). Modal classify–analyze assignments did not propagate profile uncertainty.

### 2.8. Sensitivity Analyses

To assess robustness to analytic choices, we conducted two prespecified sensitivity analyses: (i) refitting after removing RASS, and (ii) respecifying the measurement model using rank-transformed Gaussian, categorical (poLCA), and independent-Poisson count mixtures. Three exploratory analyses were also conducted: comparison of alternative Gaussian covariance structures (variable-volume, variable-shape diagonal (VVI); variable-volume, variable-shape full (VVV)), refitting on standardized (z-scored) inputs, and comparison against naïve median-split classifications. Agreement between each refit and the primary K = 4 solution was quantified by the adjusted Rand index (ARI).

### 2.9. Reproducibility and Software

All analyses were performed in R (version 4.3.0). We used mclust (version 6.1.1) for latent profile analysis and the adjusted Rand index; lme4 (version 1.1-35.5) for mixed-effects logistic regression (GLMM); ordinal (version 2023.12-4.1) for cumulative-link mixed models (CLMM); poLCA (version 1.6.0.1) and flexmix (version 2.3-20) for the categorical and independent-Poisson sensitivity analyses; and multilevLCA (version 2.0.1) for the two-level latent class sensitivity analysis (Section 3.8). All tests were two-sided with α = 0.05.

## 3. Results

### 3.1. Cohort Characteristics

**Figure 1:**
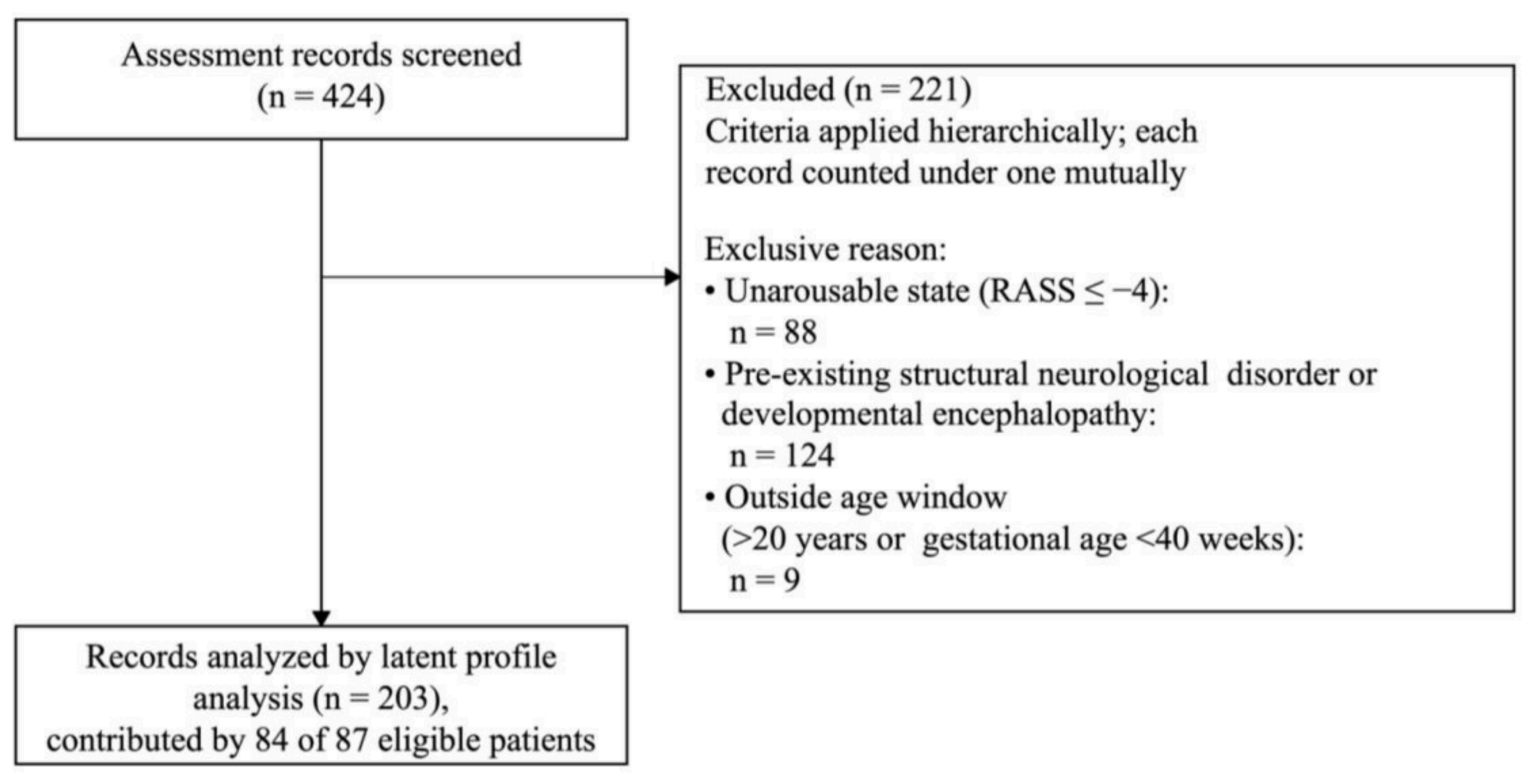
Participant flow (Strengthening the Reporting of Observational Studies in Epidemiology [STROBE] diagram). Of 424 assessment records screened, 221 were excluded; the exclusion criteria were applied hierarchically in the order listed, so that each excluded record was counted under a single, mutually exclusive reason (unarousable state, Richmond Agitation–Sedation Scale [RASS] ≤ −4, *n* = 88; pre-existing structural neurological disorder or developmental encephalopathy, *n* = 124; age outside the inclusion window, >20 years or gestational age < 40 weeks, *n* = 9), leaving 203 assessment records contributed by 84 of 87 eligible patients for the latent profile analysis.

Of 424 assessment records screened, 221 were excluded under the prespecified, hierarchically applied criteria. Each record was attributed to a single, mutually exclusive reason: an unarousable state (RASS ≤ −4; *n* = 88), a pre-existing structural neurological disorder or developmental encephalopathy (*n* = 124), or age outside the inclusion window (>20 years or gestational age < 40 weeks; *n* = 9). The final analytic set comprised 203 records, contributed by 84 of 87 eligible patients—the remaining three contributed no qualifying record—with complete data on all four input variables (Figure 1).

Cohort characteristics are summarized in Table 1.

### 3.2. Class Enumeration and Model Selection

**Figure 2:**
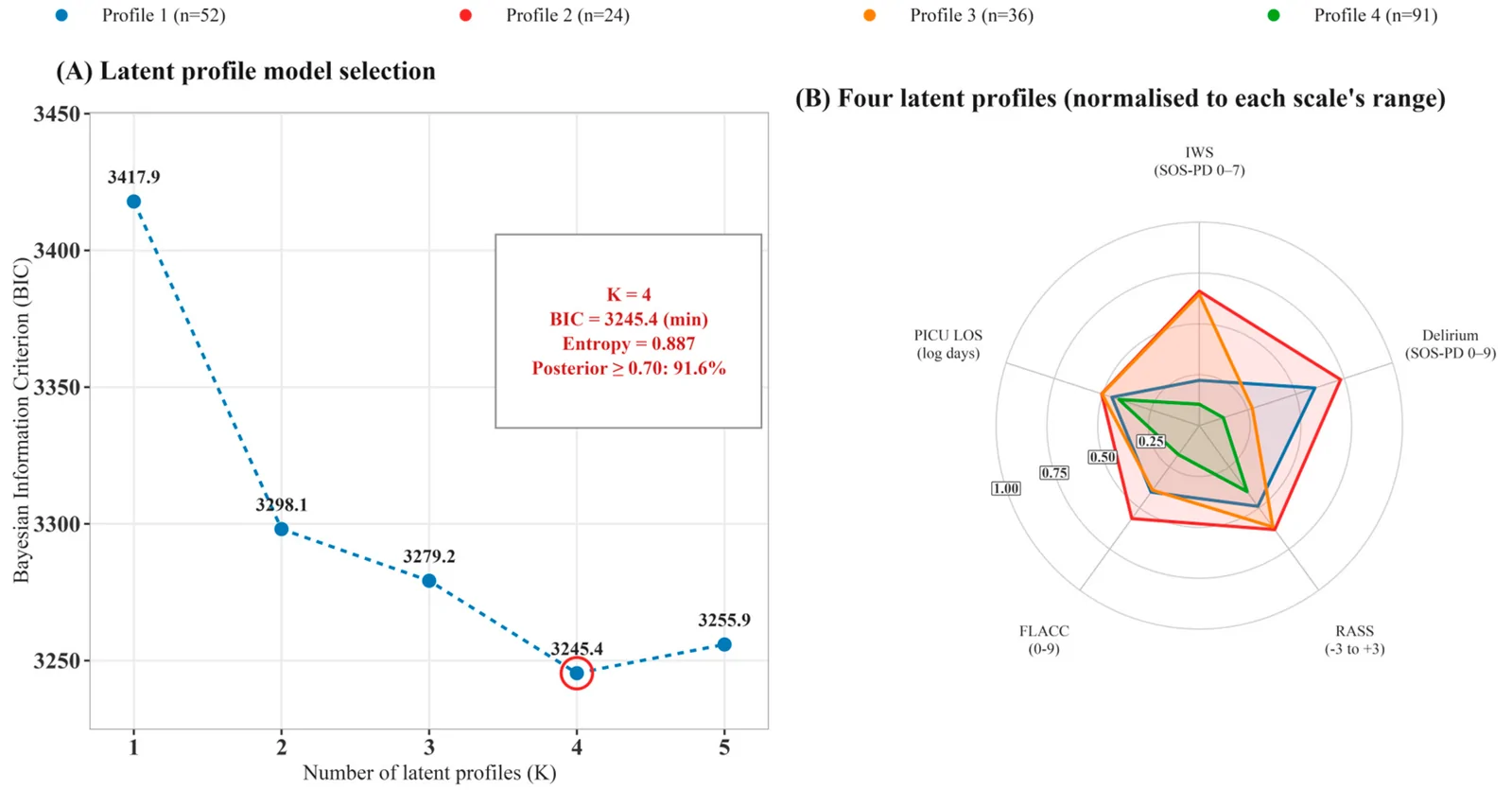
Model selection and latent profile structure. (**A**) Bayesian information criterion (BIC) across latent profile solutions (K = 1–5), with the BIC-minimizing four-profile solution highlighted. (**B**) Radar chart of the four profile means on five axes: the iatrogenic withdrawal syndrome (IWS) and delirium subscores of the Sophia Observation withdrawal Symptoms–Paediatric Delirium (SOS-PD) scale, the Richmond Agitation–Sedation Scale (RASS), the Face, Legs, Activity, Cry, Consolability (FLACC) scale, and pediatric intensive care unit (PICU) length of stay. Each axis was normalized to its within-sample range (0–1); PICU length of stay is shown for context and was not a primary profiling input.

We evaluated K = 1–5 using BIC, entropy, and the proportion with modal posterior ≥ 0.70. BIC was lowest at K = 4 (3417.9, 3298.1, 3279.2, 3245.4, 3255.9), where confident assignment peaked at 91.6%. Entropy was adequate (0.887; threshold 0.80), although entropy and BLRT marginally favored K = 5 (with the caveat that BLRT may over-extract) [24]. At K = 3, Profiles 1 and 2 merged; at K = 5, the additional class was a small Profile 2 fragment (*n* = 9). We retained K = 4 based on these criteria, parsimony, interpretability, and re-implementation (ARI = 0.9225; Supplementary Table S1). This primary-specification partition was not robust across alternative measurement-model specifications (Figure 2; Section 3.7).

### 3.3. Latent Class Structure

**Figure 3:**
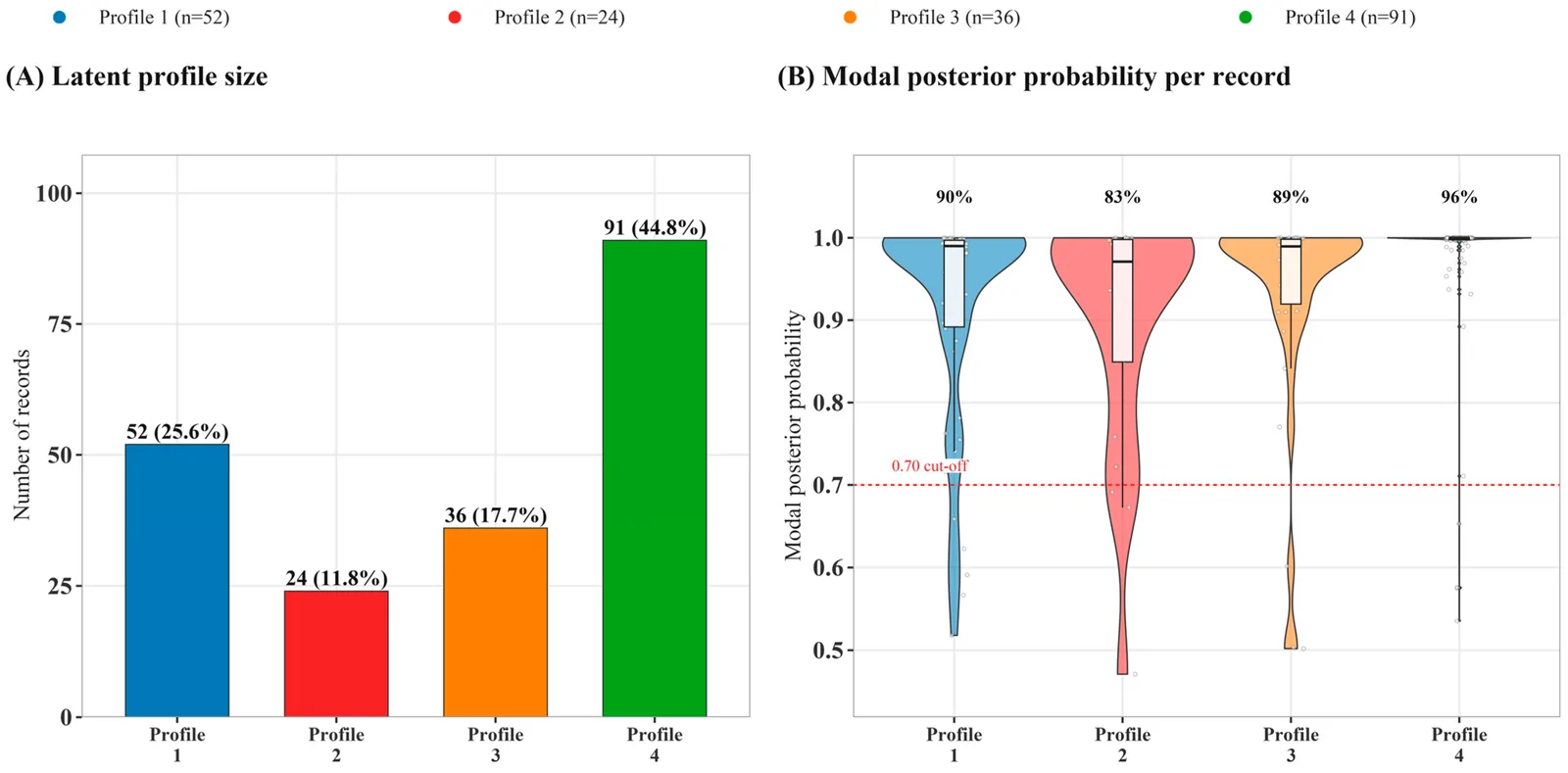
Latent profile sizes and classification certainty (*n* = 203). (**A**) Number of records per latent profile, with the percentage of all records shown alongside each count. (**B**) Distribution of modal posterior probability per record by profile, shown as violin plots with an inner box plot (median and interquartile range) and jittered individual records; the red dashed line marks the 0.70 classification cut-off, and the value above each profile is the percentage of its records assigned with modal posterior ≥ 0.70 (Profile 1, 90%; Profile 2, 83%; Profile 3, 89%; Profile 4, 96%; overall 91.6%).

The retained four-class solution partitioned the 203 records into groups of unequal size: Profile 1 (*n* = 52), Profile 2 (*n* = 24), Profile 3 (*n* = 36), and Profile 4 (*n* = 91); the distribution and posterior-probability assignment are displayed in Figure 3.

### 3.4. Class Profiles and Separation

**Figure 4:**
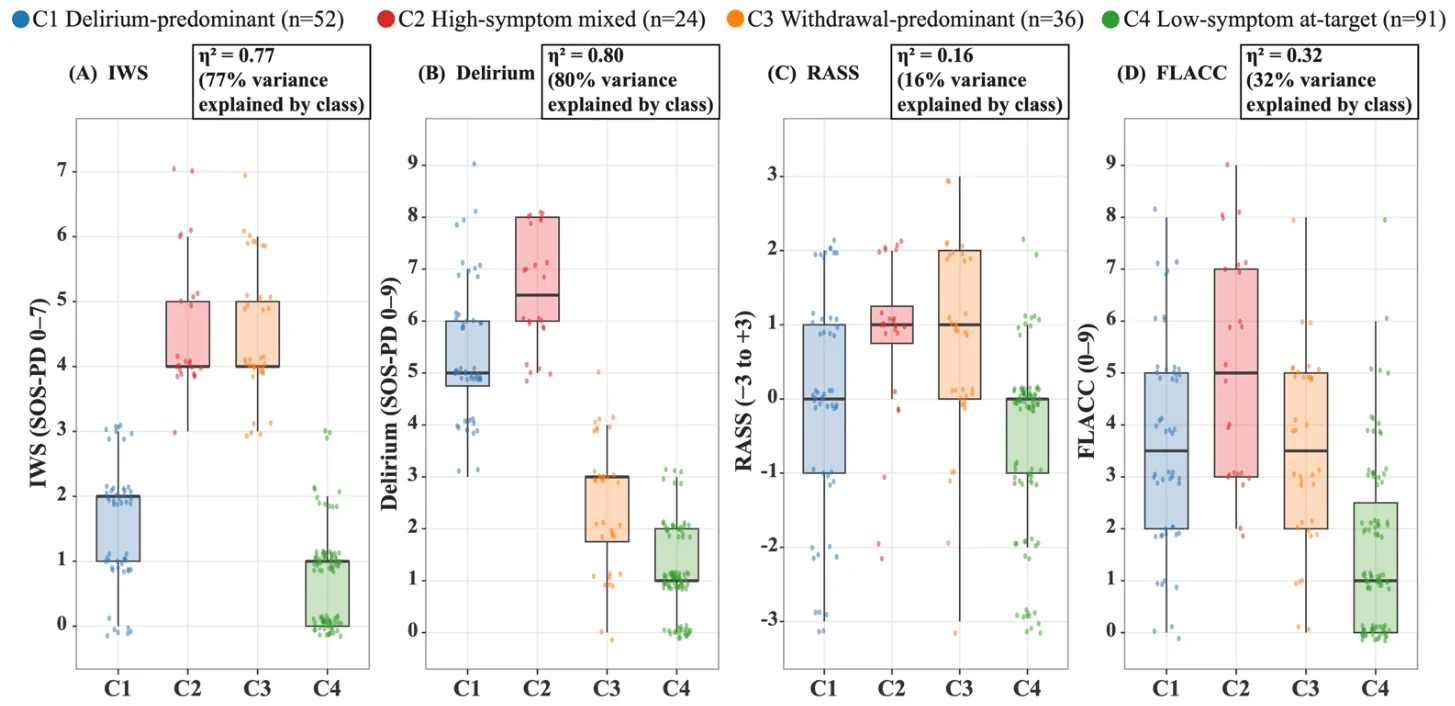
Profile-wise distributions of the four bedside scores—iatrogenic withdrawal syndrome (IWS), delirium, Richmond Agitation–Sedation Scale (RASS), and Face, Legs, Activity, Cry, Consolability (FLACC)—across the four latent profiles (*n* = 203), shown as per-profile box plots (median, interquartile range [IQR], and whiskers) overlaid with jittered raw observations. Each score is plotted on its bounded, discrete support so that all mass lies within the instrument’s legal range. η^2^ above each panel is the between-profile variance explained.

The four profiles differed most on the delirium and withdrawal subscores and least on sedation and pain. Profile-level means and dispersion (mean ± SD, with η^2^ as the overall measure of separation) are reported in Table 2 and displayed in Figure 4. Because they were computed on the indicators that defined the partition, η^2^ and the pairwise Cohen’s d quantify within-model descriptive separation on the input scales rather than external validation of distinct conditions. Separation was largest on the two most-separating scales: η^2^ = 0.798 (95% confidence interval (CI) 0.760–0.839) for the SOS-PD delirium subscore and 0.773 (95% CI 0.730–0.817) for the SOS-PD-IWS. By comparison, η^2^ was 0.320 (95% CI 0.215–0.443) for FLACC and 0.157 (95% CI 0.091–0.259) for RASS. The Kruskal–Wallis test was significant for every scale (SOS-PD-IWS *p* < 0.0001; delirium subscore *p* < 0.0001; RASS *p* < 0.0001; FLACC *p* < 0.0001) and borderline for PICU length of stay (*p* = 0.0547).

The central contrast was between Profile 1 and Profile 3. The former combined a high delirium score (5.38 ± 1.32) with a low withdrawal score (1.56 ± 0.92), whereas the latter showed the inverse (withdrawal 4.53 ± 1.06; delirium 2.47 ± 1.25). Separation between them was large on both decisive scales (absolute Cohen’s |d| = 3.05, 95% CI 2.42–3.66, for the SOS-PD withdrawal subscore and |d| = 2.26, 95% CI 1.71–2.79, for the delirium subscore). Because these are the input subscores themselves, the magnitudes describe divergence on the input scales, not independent diagnostic discrimination. Profile 2 showed simultaneously elevated delirium (6.58 ± 1.14) and withdrawal (4.62 ± 1.06) subscores with the highest pain score (FLACC 5.08 ± 2.19). Profile 4 had low scores across all scales (delirium subscore 1.12 ± 0.84; SOS-PD-IWS 0.74 ± 0.77), consistent with a comfortable, at-target state. Profile-wise PICU length of stay is in Table 1.

Class separation is summarized by η^2^ from a one-way analysis of variance (ANOVA) on each indicator using profile membership as the grouping factor, with 95% CIs from a record-level bootstrap (B = 2000); η^2^ ≥ 0.14 is conventionally considered large. Parenthetical ranges in the variable column are the cohort-observed ranges, not each instrument’s full possible score range. Pairwise standardized mean differences (Cohen’s d) for the profile contrasts are reported as absolute values |d|, with 95% CIs, in the main text. The PICU length-of-stay difference across profiles (Table 1) was borderline by the Kruskal–Wallis test (*p* = 0.0547).

### 3.5. Medication and Respiratory-Support Distribution

**Figure 5:**
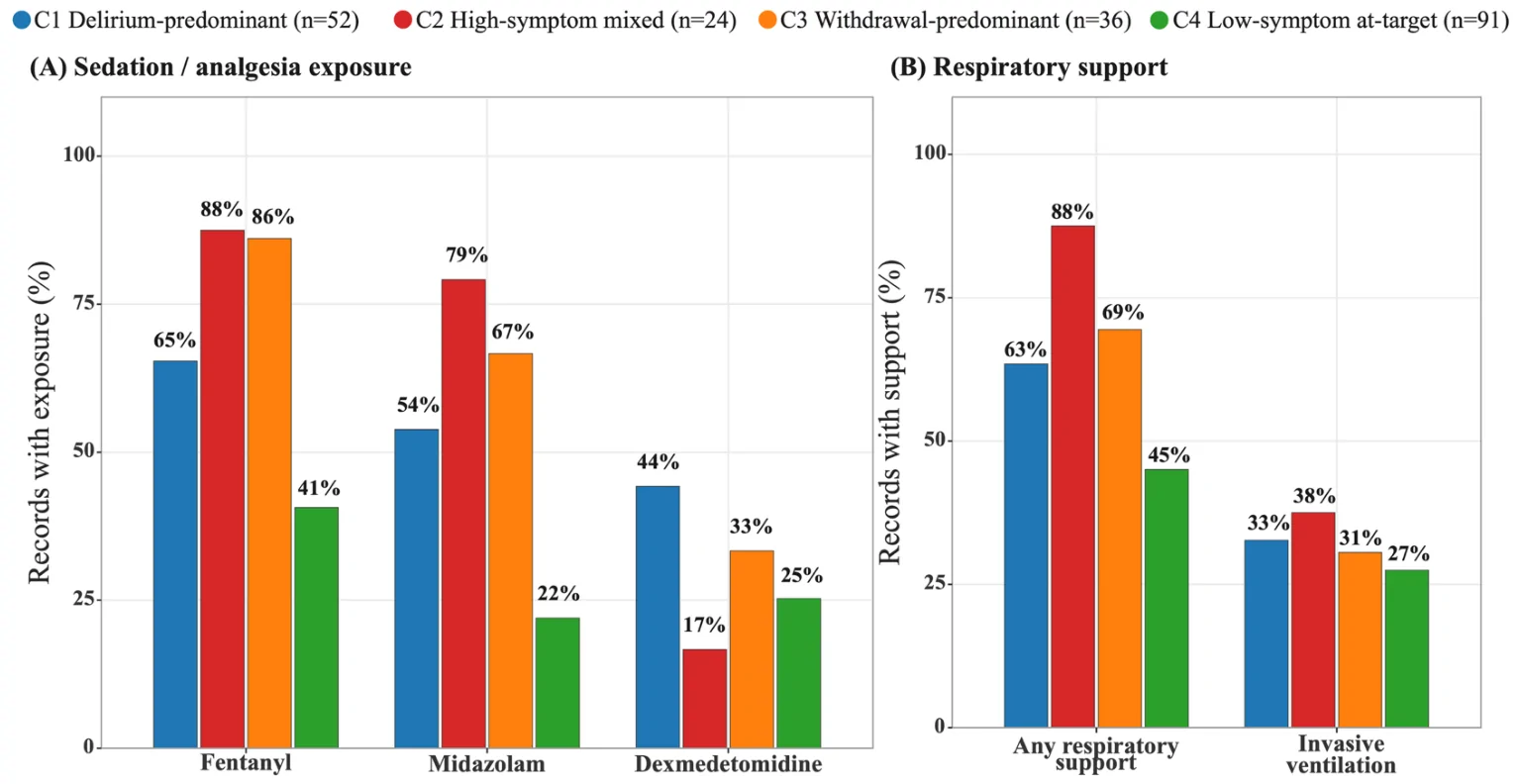
Medication and respiratory-support use across four profiles (*n* = 203). (**A**) Sedation/analgesia exposure (fentanyl, midazolam, dexmedetomidine). (**B**) Respiratory support (any respiratory support, invasive ventilation).

Medication and respiratory support differed across profiles (Table 3; Figure 5). Associations were strongest for fentanyl (χ^2^(3) = 32.74, *p* < 0.0001; Cramér’s V = 0.402) and midazolam (χ^2^(3) = 39.30, *p* < 0.0001; V = 0.440). They were weaker for dexmedetomidine (χ^2^(3) = 8.09, *p* = 0.0441; V = 0.200) and intermediate for respiratory support (χ^2^(3) = 17.44, *p* = 0.0006; V = 0.293). Intubation did not differ (χ^2^(3) = 1.07, *p* = 0.7850; V = 0.072). Six of 30 Bonferroni-corrected contrasts remained significant, all involving symptomatic profiles versus Profile 4; none involved dexmedetomidine or intubation (Table 3; Supplementary Table S3). The remaining 24 were non-significant: all 30 values are available in the analysis repository.

Omnibus chi-squared and Cramér’s V are reported for each binary variable; Bonferroni-significant pairwise contrasts (per-comparison α = 0.00167; family α = 0.05 over 30 contrasts) are summarized in the last column, using the numeric profile labels defined in Table 1 (Profile 1 = Delirium-predominant, Profile 2 = High-symptom mixed, Profile 3 = Withdrawal-predominant, Profile 4 = Low-symptom at-target). Per-cell *n* (%) by profile are provided in Supplementary Table S3. Abbreviation: df, degrees of freedom.

### 3.6. Adjusted Analysis

Adjusted analyses were descriptive (Section 2.7). Fentanyl was associated with Profile 3 (adjusted OR 5.25, 95% CI 1.80–15.26, *p* = 0.0024) and dexmedetomidine with Profile 1 (adjusted OR 2.62, 95% CI 1.26–5.46, *p* = 0.010). Midazolam was associated with a higher SOS-PD-IWS category (proportional-odds OR 4.51, 95% CI 2.47–8.25, *p* < 0.0001), not a continuous change. Hard modal assignment (91.6% posterior ≥ 0.70) ignored classification uncertainty. Susceptibility may vary (e.g., trisomy 21 reduces midazolam’s sedative effect after pediatric cardiovascular surgery) [27]. Concurrent exposure and profile measurement preclude causal inference because of possible confounding by indication or reverse causality.

### 3.7. Sensitivity Analysis

Removing RASS, the weakest separator (η^2^ = 0.157), again selected four classes by BIC, with nearly identical sizes (54/24/34/91) and assignments (ARI = 0.96). Because RASS ≤ −4 records were excluded, this tests RASS as an input only within the observed sedation range.

Class number depended on the measurement model. Only tied-diagonal EEI selected K = 4 by BIC; untied VVI and full VVV selected K = 2. When forced to K = 4, ARIs were 0.520 and 0.354. Categorical and independent-Poisson models also selected two classes (ARIs 0.475 and 0.346). Rank-transformed Gaussian selected K = 5 (ARI 0.56). Thus, competing models showed only partial similarity in record grouping (ARI = 0.346–0.560) and did not reproduce the finer four-profile assignments.

This was not a raw-scale weighting artifact. Although SOS-PD subscores contributed 57.5% of raw variance, standardized inputs again selected K = 4 and almost reproduced assignments (ARI 0.977). Equal-variance weighting therefore preserved the structure; the tied-diagonal covariance assumption remained influential. Supplementary Tables S1 and S2 report all comparisons.

Naïve classifications also agreed poorly: ARI 0.389 for 16 median-split cells and 0.495 for four groups based on the two SOS-PD subscores. Only 24 of 72 both-high records matched Profile 2 (positive predictive value 33.3%). Thus, simple thresholds did not reproduce the profiles.

### 3.8. Patient-Level Structure and Robustness

Patient-level identifiers obtained from the participating institution after the primary analysis allowed the partition to be examined for within-patient structure. The 203 records came from 84 patients (median 1, maximum 12 records per patient; record-to-patient ratio 2.42). Among the 39 patients who contributed more than one record, 21 (53.8%) had records assigned to more than one profile. Four patients, including the two highest record-contributors, spanned all four profiles. This pattern is consistent with the profiles reflecting time-varying bedside states rather than fixed patient attributes.

Patient-level stability was assessed in three ways, with only moderate, not strong, agreement. A patient-cluster bootstrap (resampling whole patients) gave an adjusted Rand index of 0.833 (95% interval 0.48–0.97); a one-record-per-patient refit gave 0.819 (95% interval 0.46–0.95); and leaving out the highest-contributing patients retained all four profiles. These wide intervals offer only partial, moderate reassurance that the four-class structure is not driven by repeated sampling of a few patients (Supplementary Note S1).

Two multilevel extensions assessed within-patient correlation. A two-level Gaussian mixture reproduced the primary solution when random-effect variance was fixed at zero (ARI 0.99), but free estimation was unstable across five starts (ARI 0.52–0.63). A categorical model on discretized indicators (multilevLCA) was non-identified, consistent with limited between-level replication [28] and distinct from classify–analyze bias [29]. Reliable multilevel estimation was therefore not feasible with 203 records from 84 patients.

## 4. Discussion

LPA of 203 PICU records identified four profiles, with Profiles 1 and 3 separated mainly by opposing SOS-PD delirium and withdrawal patterns; sedation and pain were descriptive. Because the same scales defined and labeled profiles, these are score patterns, not reference-standard diagnoses. Joint review of the underlying scores may summarize discordant signals and prompt reassessment, consistent with multidomain guidance [2,3], but the profiles themselves should not be used in clinical care before prospective external validation.

LCA has identified outcome-associated ARDS [13], sepsis [14], adult delirium [6], and post-operative [7] subgroups, but not the pediatric delirium–withdrawal boundary. Unlike those externally validated phenotypes, ours use the same behavioral scales for derivation and description and lack equivalent prognostic standing. This appears to be the first PICU bedside application; weak agreement with median-split rules, including identification of only one-third of Profile 2, indicates that the model identified patterns not reproduced by simple threshold rules (Section 3.7).

Before external prospective validation, Profiles 1 and 3 should not be used in clinical care, including for diagnosing delirium or IWS, selecting drugs, or setting weaning schedules. A future validation study could evaluate whether displaying the four scores together and flagging discordant delirium–withdrawal patterns improves bedside reassessment, independently adjudicated diagnoses, or outcomes. Multicenter, prospective testing must establish accuracy, reproducibility, and clinical utility before implementation.

Japanese SOS-PD delirium and withdrawal components are validated in Japanese cohorts [17,18], and the original instrument in a multicenter cohort [30]. However, CAPD [8], pCAM-ICU [9], and psCAM-ICU [10,11] use different items and age ranges; these profile definitions cannot be transferred directly. Their broader multidomain concept requires instrument-specific re-derivation and external validation, including an appropriate withdrawal measure. Prespecified features, multiple selection criteria, and re-implementation addressed model and input sensitivity [31].

A substantially larger, preferably multicenter, cohort—with sufficient patients rather than records—would allow a multilevel mixture model to partition within- from between-patient variation directly and test whether the delirium- and withdrawal-predominant patterns persist once patient-level dependence is fully accounted for.

Single-center, retrospective, observational data from a small tertiary PICU using the Japanese language SOS-PD limit external validity. Most screened records were excluded for structural neurological disorder or unarousable RASS ≤ −4 (Figure 1); excluded-record baselines were unavailable, precluding formal assessment of selection bias. This arousable-patient enrichment limits applicability to deep-sedation subgroups (post-cardiac-surgery, extracorporeal membrane oxygenation (ECMO), prolonged sedation); the RASS leave-out analysis (Section 3.7) covers only this range. Profiles link only to bedside variables, not independent outcomes; length of stay is context only, and outcome-linkage remains future work.

Records cluster within patients, so effective sample size is smaller than record count, overstating precision of between-class contrasts and BIC enumeration. Prespecified multilevel extensions (Section 3.8) were not identifiable at 84 patients/203 records (continuous or categorical), so assessment-level findings are reported, with patient-level resampling giving only partial, moderate reassurance. No fixed minimum-sample rule exists for LPA, and the smallest class (*n* = 24) had a wide bootstrap interval and remains provisional.

Medication models used hard assignments, so associations remain descriptive. Restricted RASS range, multiplicity correction, and interrater variability [32] may have obscured differences. Reporting followed the GRoLTS checklist [33]. Only tied-diagonal EEI selected K = 4; untied, categorical, and count models favored two classes and only partly recovered assignments (Section 3.7), consistent with the sensitivity of latent class solutions to clustering and distributional assumptions [34,35]. Profiles 1–3 remain provisional; Profile 4 was more stable. Ordinal or count-mixture replication is needed (Supplementary Table S2).

## 5. Conclusions

Routine PICU scales contained model-specific candidate delirium-predominant (Profile 1) and withdrawal-predominant (Profile 3) score patterns. Without an independent reference standard, the four profiles are not diagnostic. Their number depended on the tied-diagonal Gaussian model, limiting generalizability. The profiles may be interpreted only as descriptive score summaries and should not be used in clinical care until prospective external multicenter validation against independent assessments and outcomes.

## Figures and Tables

**Table 1:** Cohort characteristics by latent profile (*n* = 203).

Characteristic	Overall (*n* = 203)	Profile 1 (Delirium-Predominant) (*n* = 52)	Profile 2 (High-Symptom Mixed) (*n* = 24)	Profile 3 (Withdrawal-Predominant) (*n* = 36)	Profile 4 (Low-Symptom at-Target) (*n* = 91)
Age, months, median [IQR]	12.0 [6.0–40.0]	13.5 [7.0–30.5]	17.5 [7.0–34.0]	32.0 [17.2–51.5]	11.0 [4.0–36.5]
Female, *n* (%)	116 (57.1)	27 (51.9)	11 (45.8)	21 (58.3)	57 (62.6)
Trisomy, *n* (%)	57 (28.1)	12 (23.1)	13 (54.2)	20 (55.6)	12 (13.2)
Developmental delay, *n* (%)	82 (40.4)	15 (28.8)	16 (66.7)	26 (72.2)	25 (27.5)
Post-operative, *n* (%)	142 (70.0)	39 (75.0)	20 (83.3)	22 (61.1)	61 (67.0)
Mechanical ventilation, *n* (%)	62 (30.5)	17 (32.7)	9 (37.5)	11 (30.6)	25 (27.5)
Any respiratory support, *n* (%)	120 (59.1)	33 (63.5)	21 (87.5)	25 (69.4)	41 (45.1)
PICU length of stay, days, median [IQR]	12.0 [5.0–28.0]	14.0 [5.0–23.0]	13.5 [6.8–53.0]	20.5 [7.0–33.2]	8.0 [4.0–22.0]

Continuous variables are summarized as median [interquartile range (IQR)]; binary variables as *n* (%) of all 203 records. Trisomy codes 1 and 2 are pooled as “any trisomy”. Mechanical ventilation refers to invasive intubation; “any respiratory support” includes invasive ventilation, bilevel positive airway pressure (BiPAP), and high-flow nasal cannula (HFNC). PICU, pediatric intensive care unit.

**Table 2:** Bedside scale profiles and profile separation (mean ± standard deviation [SD]; *n* = 203). Abbreviations: CI, confidence interval; FLACC, Face, Legs, Activity, Cry, Consolability; IQR, interquartile range; IWS, iatrogenic withdrawal syndrome; LOS, length of stay; PICU, pediatric intensive care unit; RASS, Richmond Agitation–Sedation Scale; SOS-PD, Sophia Observation withdrawal Symptoms–Paediatric Delirium.

Variable	Profile 1 (Delirium-Predominant) (*n* = 52)	Profile 2 (High-Symptom Mixed) (*n* = 24)	Profile 3 (Withdrawal-Predominant) (*n* = 36)	Profile 4 (Low-Symptom at-Target) (*n* = 91)	η^2^ (95% CI)
SOS-PD withdrawal subscore (SOS-PD-IWS; 0–7)	1.56 ± 0.92	4.62 ± 1.06	4.53 ± 1.06	0.74 ± 0.77	0.773 [0.730–0.817]
SOS-PD delirium subscore (0–9)	5.38 ± 1.32	6.58 ± 1.14	2.47 ± 1.25	1.12 ± 0.84	0.798 [0.760–0.839]
RASS (−3 to +3)	−0.06 ± 1.51	0.79 ± 1.14	0.69 ± 1.33	−0.59 ± 1.22	0.157 [0.091–0.259]
FLACC (0–9)	3.63 ± 1.97	5.08 ± 2.19	3.53 ± 1.83	1.58 ± 1.65	0.320 [0.215–0.443]
PICU LOS, days (median [IQR])	14.0 [5.0–23.0]	13.5 [6.8–53.0]	20.5 [7.0–33.2]	8.0 [4.0–22.0]	—

**Table 3:** Medication and respiratory-support use by profile (*n* = 203).

Variable	χ^2^ (df = 3)	*p*	Cramér V	Bonferroni-Significant Pairs
Fentanyl, any use	32.74	<0.0001	0.402	Profile 2 vs. Profile 4; Profile 3 vs. Profile 4
Midazolam, any use	39.30	<0.0001	0.440	Profile 1 vs. Profile 4; Profile 2 vs. Profile 4; Profile 3 vs. Profile 4
Dexmedetomidine, any use	8.09	0.0441	0.200	none
Any respiratory support	17.44	0.0006	0.293	Profile 2 vs. Profile 4
Intubation (invasive)	1.07	0.7850	0.072	none

## Data Availability

The data presented in this study are available on request from the corresponding author due to privacy and ethical restrictions. The de-identified dataset with analysis variables is held by the corresponding author. Researchers interested in accessing the data for future studies may contact the corresponding author (Yujiro Matsuishi, matsuishi.yujiro.xa@alumni.tsukuba.ac.jp).

## Supplementary Materials

The following supporting information can be downloaded at: https://www.mdpi.com/article/10.3390/jcm15166134/s1. Table S1. Adjusted Rand index reconciliation against the original class labels in the analysis dataset. Table S2. Measurement-model and indicator-set sensitivity analyses, with comparison against naïve subscore-split rules. Table S3. Per-cell *n* (%) for medication and respiratory-support variables, with uncorrected and Bonferroni-corrected pairwise p-values; Note S1. Patient-level structure and robustness. Note S2. Reproducibility environment and statistical methods detail. Note S3. Reporting checklists; Strengthening the Reporting of Observational Studies in Epidemiology (STROBE) Checklist (Cohort Variant); Guidelines for Reporting on Latent Trajectory Studies (GRoLTS) Checklist.
