# Supplementary material for: Delirium and Iatrogenic Withdrawal Profiles in the Pediatric Intensive Care Unit (PICU): A Latent Profile Analysis of Nurse-Administered Bedside Scales
Source: J Clin Med. 2026 Aug 7;15(16):6134. doi: 10.3390/jcm15166134 (PMC13513878; doi:10.3390/jcm15166134)
Supplement: Supplementary file 1 [file jcm-15-06134-s001.zip › jcm-4427873-supplementary.pdf]

Supplementary Materials

Supplementary Materials for: "Delirium and Iatrogenic Withdrawal Profiles in the Pediatric Intensive Care Unit (PICU): A Latent Profile Analysis of Nurse-Administered Bedside Scales."

Table S1. Adjusted Rand index reconciliation against the original class labels in the analysis dataset

Re-estimated K = 4 assignments were compared with the original labels.

Confusion matrix (re-estimated semantic-aligned label vs original class_id)

	original class_id = 1	= 2	= 3	= 4
Re-estimated = 1	48	4	0	0
Re-estimated = 2	0	24	0	0
Re-estimated = 3	1	1	32	2
Re-estimated = 4	0	0	0	91

Summary

- **Adjusted Rand index** (mclust::adjustedRandIndex): **0.9225**.
- **Discordant records:** 8 of 203 (3.9%), in the mid-range of the posterior-probability distribution.
- The 50-start solution had higher log-likelihood; the eight disagreements reflected the original lower-likelihood fit, not label switching.

Table S2. Measurement-model and indicator-set sensitivity analyses, with comparison against naïve subscore-split rules

Rank-transformed EEI with 50 random starts selected K = 5 by BIC; constrained K = 4 gave ARI 0.5558 versus the primary partition [29,30].

Measurement-level and indicator-set sensitivity refits

Each RASS-excluded or alternative measurement-model refit reselected K by BIC and was compared with primary K = 4 by ARI.

Specification	BIC-selected K	ARI vs primary EEI-K4	Min class size or note
Primary raw Gaussian EEI	4	1.000	min 24 (52/24/36/91)
Standardised (z) Gaussian EEI	4	0.977	K = 4 partition essentially unchanged
Untied diagonal (VVI)	2	0.520	min 72 at K = 2
Class-specific full (VVV)	2	0.354	min 76 at K = 2
Rank-transformed EEI	5	0.56	—
Categorical LCA Scheme A (poLCA 4-level)	2	0.475	—
Categorical LCA Scheme B (poLCA 3-level)	2	0.466	—
Independent-Poisson mixture	2	0.346	—
RASS-excluded 3-var EEI	4	0.960	—

Only EEI selected K = 4. Standardised EEI (ARI 0.977) and RASS-excluded EEI (0.960) preserved four profiles; other specifications selected K = 2 or had low ARI. C4 was most stable.

Added value over naïve subscore-split rules

Two naïve classifications showed poor agreement with primary EEI K = 4.

Naïve rule	Number of groups	ARI vs primary EEI-K4
16-cell median split (all four inputs)	16	0.389
4-group SOS-PD-subscore median-split rule	4	0.495

Of 72 both-subscores-high records, 24 were C2 (positive predictive value 33.3%); C2 was jointly high across all four inputs.

Table S3. Per-cell n (%) for medication and respiratory-support variables, with uncorrected and Bonferroni-corrected pairwise p-values

Values are n (% within profile). Omnibus 4 × 2 χ^2 tests report Cramér's V; 30 pairwise Fisher tests (5 variables × 6 pairs) use Bonferroni α = 0.00167 (family α = 0.05). C1–C4 sizes are 52, 24, 36, and 91.

Per-cell n (% within profile) and omnibus test

Variable	C1	C2	C3	C4	$\chi^2(3)$	*p*	Cramér's *V*
Fentanyl use	34/52 (65.4)	21/24 (87.5)	31/36 (86.1)	37/91 (40.7)	32.74	3.66×10^{-7}	0.402
Midazolam use	28/52 (53.8)	19/24 (79.2)	24/36 (66.7)	20/91 (22.0)	39.30	1.50×10^{-8}	0.440
Dexmedetomidine use	23/52 (44.2)	4/24 (16.7)	12/36 (33.3)	23/91 (25.3)	8.09	0.0441	0.200
Any respiratory support	33/52 (63.5)	21/24 (87.5)	25/36 (69.4)	41/91 (45.1)	17.44	5.74×10^{-4}	0.293
Invasive mechanical ventilation	17/52 (32.7)	9/24 (37.5)	11/36 (30.6)	25/91 (27.5)	1.07	0.7850	0.072

Pairwise Fisher exact tests — the six Bonferroni-significant contrasts (of 30)

Variable	Contrast	*p* (uncorrected)	*p* (Bonferroni)
Fentanyl use	C2 vs C4	5.14×10^{-5}	0.00154
Fentanyl use	C3 vs C4	3.12×10^{-6}	9.35×10^{-5}
Midazolam use	C1 vs C4	1.91×10^{-4}	0.00572
Midazolam use	C2 vs C4	3.92×10^{-7}	1.18×10^{-5}
Midazolam use	C3 vs C4	3.51×10^{-6}	1.05×10^{-4}
Any respiratory support	C2 vs C4	1.74×10^{-4}	0.00523

Of 30 corrected contrasts, six—all symptomatic profiles versus C4—were significant; the other 24 were non-significant (Bonferroni $p > 0.05$).

Supplementary Note S1. Patient-level structure and robustness

Identifiers linked 203 records to 84 patients (median 1 record, maximum 12; ratio 2.42); 45 patients contributed one record.

Within-patient structure

Of 39 multi-record patients, 21 (53.8%) crossed profiles; the four highest contributors crossed all four, consistent with time-varying states.

Patient-level robustness

1. Patient-cluster bootstrap: ARI 0.833 (95% percentile interval 0.48–0.97).
2. One-record-per-patient refit: ARI 0.819 (0.46–0.95).
3. Excluding high-contributing patients retained all four profiles.

These analyses argue against a repeated-sampling artifact.

Operational consequences and limitations

1. Primary statistics use PICU assessment records.
2. Clustering (203 records/84 patients) reduces effective sample size and may overstate precision of contrasts and BIC enumeration; sensitivity analyses provide moderate reassurance.

Supplementary Note S2. Reproducibility environment and statistical methods detail

Supplementary statistical methods

- BLRT (cf. §2.4): 999 mclust::mclustBootstrapLRT replicates; supplementary because finite samples may over-extract.
- Pairwise (cf. §2.5): Mann–Whitney U/rank-biserial r; 4 × 2 χ^2/Cramér's V then Fisher exact; 30 contrasts used Bonferroni $\alpha = 0.00167$, with raw and corrected p values.
- Adjusted models (cf. §2.5): patient random intercept, age, post-operative status, developmental delay, trisomy, and respiratory support; one-vs-rest logistic GLMM for profiles and proportional-odds CLMM for score levels.

Primary analysis (authoritative)

- R 4.3.0 on aarch64-apple-darwin20.
- Inputs: SOS-PD-IWS, SOS-PD delirium, RASS, and FLACC.
- Authoritative script: analysis/SOS_PD_analysis.R.

Key R packages: mclust 6.1.1, readr 2.1.5, dplyr 1.1.4, tidyr 1.3.1, effsize 0.8.1, ggplot2 3.5.2.

Convergence checks

- At K = 4, 50-start EM reached loglik = −1561.60 (BIC = 3245.4), identical with 200 and 500 starts.
- Random starts avoided a suboptimal default mclust solution at G = 4; tied-diagonal EEI was prespecified for parsimony.

Supplementary Note S3. Reporting checklists

Separate STROBE [16] and GRoLTS [33] checklists map each item to the manuscript and mark Yes/Partial/N/A.
